# The Editor's Letter-Box

**Published:** 1917-03-31

**Authors:** Arthur Stanley, E. Cooper Perry

**Affiliations:** The College of Nursing, Ltd., 83 Pall Mall, London, S.W.; The College of Nursing, Ltd., 83 Pall Mall, London, S.W.


[Copy.]

Royal British Nurses' Association and College of Nursing, Ltd.

Dear Mr. Pitt,—I have now had an opportunity of discussing the matter with Sir Cooper Perry, and we agree to give the undertaking asked for in your letter of 30th ultimo, on the understanding contained in the last paragraph of the same letter.

As a proof of our joint acceptance of the above terms, I have asked Sir Cooper Perry to sign with me, and his signature is appended.—I am, dear Mr. Pitt, yours faithfully,

(Signed) Arthur Stanley.
E. Cooper Perry.

The College of Nursing, Ltd.,
83 Pall Mall, London, S.W., December 11, 1916.
Messrs. Pontifex, Pitt and Co.

